# Supplementary material for: HIVprotI: an integrated web based platform for prediction and design of HIV proteins inhibitors
Source: J Cheminform. 2018 Mar 9;10:12. doi: 10.1186/s13321-018-0266-y (PMC5845081; doi:10.1186/s13321-018-0266-y)
Supplement: Supplementary file 2 — Additional file 2. Source code of HIVProtI web server. [file 13321_2018_266_MOESM2_ESM.zip › HIVprotI_Source-code/hivprotI/weka-3-6-6/doc/weka/gui/visualize/package-tree.html]

weka.gui.visualize Class Hierarchy


---


|  |  |  |
| --- | --- | --- |
| **Overview** \| **Package** \| Class \| **Tree** \| **Deprecated** \| **Index** \| **Help** \| **Weka's home** | |  |
| **PREV**   **NEXT** | **FRAMES**    **NO FRAMES**     **All Classes** |  |


---


## Hierarchy For Package weka.gui.visualize

**Package Hierarchies:**: All Packages

---

## Class Hierarchy

- java.lang.Object
  - weka.gui.visualize.**AttributePanelEvent**- java.awt.Component (implements java.awt.image.ImageObserver, java.awt.MenuContainer, java.io.Serializable)
      - java.awt.Container
        - javax.swing.JComponent (implements java.io.Serializable)
          - javax.swing.JPanel (implements javax.accessibility.Accessible)
            - weka.gui.visualize.**ClassPanel**- weka.gui.visualize.**MatrixPanel**- weka.gui.visualize.**Plot2D**- weka.gui.visualize.**PrintablePanel** (implements weka.gui.visualize.PrintableHandler)
                    - weka.gui.visualize.**VisualizePanel**
                      - weka.gui.visualize.**ThresholdVisualizePanel**
            - javax.swing.JScrollPane (implements javax.accessibility.Accessible, javax.swing.ScrollPaneConstants)
              - weka.gui.visualize.**AttributePanel**- weka.gui.visualize.**LegendPanel**
      - java.awt.Graphics
        - java.awt.Graphics2D
          - weka.gui.visualize.**PostscriptGraphics**
        - weka.gui.visualize.**JComponentWriter**
          - weka.gui.visualize.**BMPWriter**- weka.gui.visualize.**JPEGWriter**- weka.gui.visualize.**PNGWriter**- weka.gui.visualize.**PostscriptWriter**
          - weka.gui.visualize.**Messages**- weka.gui.visualize.**PlotData2D**- weka.gui.visualize.**PrintableComponent** (implements weka.gui.visualize.PrintableHandler)- weka.gui.visualize.**VisualizePanelEvent**- weka.gui.visualize.**VisualizeUtils**

## Interface Hierarchy

- weka.gui.visualize.**AttributePanelListener**- weka.gui.visualize.**Plot2DCompanion**- weka.gui.visualize.**PrintableHandler**- weka.gui.visualize.**VisualizePanelListener**

---


|  |  |  |
| --- | --- | --- |
| **Overview** \| **Package** \| Class \| **Tree** \| **Deprecated** \| **Index** \| **Help** \| **Weka's home** | |  |
| **PREV**   **NEXT** | **FRAMES**    **NO FRAMES**     **All Classes** |  |


---
